# Influence of Moxifloxacin on Hepatic Redox Status and Plasma Biomarkers of Hepatotoxicity and Nephrotoxicity in Rat

**DOI:** 10.1155/2015/192724

**Published:** 2015-10-13

**Authors:** Ayokanmi Ore, Ebenezer Tunde Olayinka

**Affiliations:** Biochemistry Unit, Department of Chemical Sciences, Ajayi Crowther University, PMB 1066, Oyo 211213, Oyo State, Nigeria

## Abstract

Moxifloxacin is a broad spectrum fluoroquinolone antibacterial agent. We examined the hepatic redox status and plasma biomarkers of nephrotoxicity and hepatotoxicity in rat following administration of moxifloxacin (MXF). Twenty-four Wistar rats, 180–200 g, were randomized into four groups (I–IV). Animals in group I (control) received 1 mL of distilled water, while animals in groups II, III, and IV received 1 mL each of MXF equivalent to 4 mg/kg b.w., 8 mg/kg b.w., and 16 mg/kg b.w., respectively. After seven days, plasma urea, bilirubin, and creatinine were significantly (*P* < 0.05) elevated in the MXF-treated animals. Activities of alkaline phosphatase, aspartate aminotransferase, and alanine aminotransferase were significantly increased in the plasma of MXF-treated animals compared to control. Also plasma total cholesterol, HDL-cholesterol, LDL-cholesterol, and triglycerides increased significantly in the MXF-treated groups relative to control. Moreover, MXF triggered a significant decrease in hepatic catalase, superoxide dismutase, and glutathione-*S* transferase activities. Likewise, MXF caused a decrease in the hepatic levels of glutathione and vitamin C. A significant increase in hepatic MDA content was also observed in the MXF-treated animals relative to control. Overall, our data suggest that the half-therapeutic, therapeutic, and twice the therapeutic dose of MXF induced nephrotoxicity, hepatotoxicity, and altered hepatic redox balance in rats.

## 1. Introduction

Moxifloxacin (MFX) is a fourth-generation synthetic fluoroquinolone antibacterial agent with a broad spectrum of bactericidal action. MXF possess enhanced activity against Gram-positive bacteria, most notably against penicillin-susceptible and penicillin-resistant strains of* S. pneumoniae*. It is available for oral and intravenous administration, respectively, as a once-daily 400 mg antibiotic for the treatment of respiratory tract infections, chronic bronchitis, and acute bacterial sinusitis and in some cases pelvic inflammatory disease, complicated and uncomplicated skin and skin structure infections, complicated intra-abdominal infections, ocular bacterial keratitis, and community acquired pneumonia [1–3].

**Figure 1:**
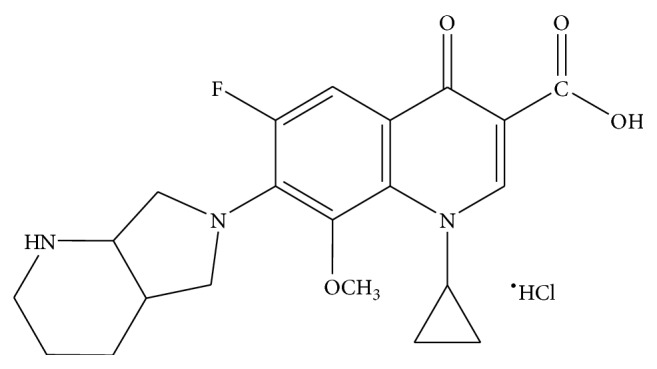
Moxifloxacin hydrochloride (1-cyclopropyl-7-[(S,S)-2,8-diazabicyclo[4.3.0]non-8-yl]-6-fluoro-8-methoxy-1,4-dihydro-4-oxo-3-quinoline carboxylic acid ^•^HCl).

MXF like other quinolones have a bicyclic aromatic core with a carbon at position 8 and demonstrate an N-1 cyclopropyl moiety (Figure 1). Following oral administration, MXF is well absorbed from the gastrointestinal tract with approximately 50% bound to serum proteins [4]. It binds weakly to plasma proteins and penetrates well into most tissue and fluid compartments. MXF is metabolized to an N-sulfate conjugate and an acyl glucuronide in humans [5]. Like other fluoroquinolones, MXF exhibit bactericidal activity by binding to bacterial topoisomerases II (DNA gyrase) and topoisomerase IV [6]. By binding to these enzymes, the fluoroquinolones interfere with DNA replication, repair, and transcription, resulting in bacterial death. The ability to target both enzymes has been promoted as a major advantage of the fluoroquinolones in preventing or delaying the emergence of resistance [7].

Most fluoroquinolones are known to be associated with some adverse effects on vital organs [8]. Previous reports suggest that free radical formation might play a role in the mechanism of some of these adverse effects [9]. Moreover, certain members of the fluoroquinolones are known to produce reactive oxygen species (ROS) in phagocytic cells [10, 11]. Side effects such as hepatotoxicity [12], phototoxicity [13], and cartilage damage [11] may be related to generation of ROS [14] and weakening of enzymatic antioxidant defence mechanism in tissues [15].

The hepatocytes like most body cells are made up of antioxidant defence system comprising nonenzymatic antioxidants including glutathione, ascorbic acid, and tocopherol and enzymatic antioxidants such as catalase, superoxide dismutase, glutathione peroxidase, and glutathione reductase [16]. They are involved in protection against tissue injuries caused by free radicals and other ROS [16]. It has been suggested that many classes of antibiotics generate varying degrees of reactive oxygen species (ROS) that contribute to cell killing [17, 18]. However, exposure to elevated level of ROS may result in weakening of cellular antioxidant capacity thereby exposing the cell to excessive level of lipid peroxidation and ultimately tissue injury [19].

Our interest in the capacity of certain fluoroquinolones to generate ROS [20, 21] prompted us to study the effects of MFX on the hepatic biomarkers of oxidative stress in rat. There is little or no previous report on the effect of MXF on the hepatic antioxidant defence system as well as markers of nephrotoxicity and hepatotoxicity. Hence, this study was designed to assess the impact of MXF on hepatic redox status as well as biomarkers of nephrotoxicity and hepatotoxicity in rat model.

## 2. Materials and Methods

### 2.1. Chemicals and Reagents

Moxifloxacin hydrochloride (Avelox) was a product of Bayer Healthcare Pharmaceutics, Leverkusen, Germany. Glutathione (GSH), 1-chloro-2,4-dinitrobenzene (CDNB), 5′,5′-dithiobis-2-nitrobenzoic acid (DTNB), thiobarbituric acid (TBA), epinephrine, and hydrogen peroxide were purchased from Sigma Chemical Company (London, UK). Assay kits for alanine aminotransferase (ALT), aspartate aminotransferase (AST), alkaline phosphatase (ALP), *γ*-glutamyl transferase (*γ*-GT), urea, creatinine, bilirubin, total cholesterol, HDL-cholesterol, LDL-cholesterol, and triglycerides were products of Randox Laboratories Ltd., Antrim, UK. All other chemicals and reagents used in this study were of analytical grade.

### 2.2. Animal Selection and Care

Twenty-four male albino rats (Wistar strain) weighing between 160 g and 180 g were used for this study. The animals were obtained from the animal holding unit of the Department of Chemical Sciences, Ajayi Crowther University, Oyo, Nigeria. The rats were acclimatised under laboratory conditions prior to the commencement of the study. The animals were housed in wire meshed cages maintained at standard conditions of temperature and humidity with an alternating light cycle (12 hr light/dark). They were fed with commercial pelletized diet (Ladokun Feeds, Ibadan, Nigeria) and supplied water* ad libitum*. The experimental protocol relating to animal handling conformed to the international guidelines on the Care and Use of Laboratory Animals [22]. 

### 2.3. Animal Grouping and Drug Administration

The rats were randomised into four experimental groups (I–IV) of 6 animals each. Group I (control) animals were administered distilled water. Animals in group II received MXF at a dose of 4 mg/kg body weight (b.w.); this is an equivalent of half of the therapeutic dose (MXF-1). Group III animals were administered 8 mg/kg b.w. MXF equivalent to the therapeutic dose used in the treatment of skin structure infection and community acquired pneumonia (MXF-2). Animals in group IV were administered MXF at 16 mg/kg b.w., equivalent to two times the therapeutic dose (MXF-3). The drug treatments lasted for 7 days.

### 2.4. Collection of Blood and Liver Samples

Blood samples were collected from each animal through retro orbitals plexus into heparinized tubes (Li heparin). Animals were thereafter euthanized and the liver was carefully excised from each animal for preparation of cytosolic fraction.

### 2.5. Preparation of Plasma and Cytosolic Fractions

Plasma was obtained by centrifugation of whole blood sample at 4000 rpm for 5 minutes using a bench centrifuge (Analytika, Athens, Greece). The plasma obtained was stored at −4°C for subsequent plasma assays. Liver samples obtained from each rat were blotted of blood stains, rinsed in ice-cold 1.15% KCl, and homogenized in 4 volumes of ice-cold 0.01 M potassium phosphate buffer (pH 7.4). The homogenates were centrifuged at 12,500 g for 15 min at −4°C in a refrigerated centrifuge (Eppendorf, Stevenage, UK) and the supernatants, termed the postmitochondrial fractions (PMF), were aliquoted and used for subsequent biochemical assays.

### 2.6. Determination of Plasma and Liver Protein Content

Protein concentration in the plasma and liver homogenate was determined by the Biuret method of Gornall et al. [23] using bovine serum albumin as standard.

### 2.7. Assay of Plasma Biomarkers of Renal Toxicity

Plasma urea and creatinine were determined with Randox diagnostic kits. Method for creatinine assays was based on colorimetric alkaline picrate methods of Jaffé [24] with creatinine-picrate complex measured at 492 nm. Plasma urea determination was based on the Fenton reaction of Tietz [25] with the Diazine chromogen formed absorbing strongly at 540 nm.

### 2.8. Assay of Plasma Biomarkers of Hepatotoxicity

Plasma total bilirubin (TBILI) determination was done using Randox diagnostic kits based on the dimethyl sulphoxide method by Tietz et al. [26]. The dimethyl sulphoxide forms a coloured compound with maximum absorption at 550 nm. Plasma alkaline phosphatase (ALP), alanine aminotransferase (ALT), and aspartate aminotransferase (AST) activities were determined using Randox diagnostic kits. ALP activity was determined in accordance with the principles of Tietz et al. [26]. The p-nitrophenol formed by the hydrolysis of p-nitrophenyl phosphate confers yellowish colour on the reaction mixture and its intensity can be monitored at 405 nm to give a measure of enzyme activity. Determination of plasma ALT and AST activities was based on the principle described by Reltman and Frankel [27]. ALT activity was measured by monitoring the concentration of pyruvate hydrazone formed with 2,4-dinitrophenylhydrazine at 546 nm. AST activity was measured by monitoring the concentration of oxaloacetate hydrazone formed with 2,4-dinitrophenylhydrazine at 546 nm.

### 2.9. Determination of Plasma Lipid Profiles

The plasma total cholesterol, HDL-cholesterol, LDL-cholesterol, and triglycerides were determined using Randox diagnostic kits and the determination was based on CHOD-PAD enzymatic colorimetric method of Trinder [28].

### 2.10. Assay for Nonenzymatic Antioxidants in the Liver

Hepatic reduced glutathione level was determined according to the method of Jollow et al. [29]. The chromophoric product resulting from the reaction of Ellman's reagent with the reduced glutathione, 2-nitro-5-thiobenzoic acid, possesses a molar absorption at 412 nm which was read in a spectrophotometer. Reduced GSH is proportional to the absorbance at 412 nm. The ascorbic acid (AA) concentration was determined according to the method of Jagota and Dani [30]. AA in biological samples reacts with Folin's reagent, an oxidizing agent, to give a blue color which has its maximum absorption at 760 nm.

### 2.11. Assay of Hepatic Antioxidant Enzymes

Hepatic glutathione-S-transferase (GST) activity was determined by the method described by Habig et al. [31] using 1-chloro-2,4-dinitrobenzene (CDNB) as substrate. The procedure of Misra and Fridovich [32] was used for the determination of hepatic superoxide dismutase (SOD) activity by measuring the inhibition of autooxidation of epinephrine at pH 10.2 and 30°C. Hepatic catalase activity was determined by the method described by Sinha [33] based on the reduction of dichromate in acetic acid to chromic acetate when heated in the presence of hydrogen peroxide (H_2_O_2_). The chromic acetate produced is measured spectrophotometrically at 570 nm.

### 2.12. Assay of Hepatic Level of Lipid Peroxidation

The extent of lipid peroxidation (LPO) in the liver was estimated by the method of Varshney and Kale [34]. The method involved the reaction between malondialdehyde (MDA; product of lipid peroxidation) and thiobarbituric acid to yield a stable pink chromophore with maximum absorption at 532 nm.

### 2.13. Statistical Analysis

Results were expressed as mean of 5 replicates ± SD. Data obtained were subjected to one-way Analysis of Variance (ANOVA) followed by Duncan multiple range test for comparison between control and treated rats in all groups using SigmaPlot Statistical application package. *P* values less than 0.05 were considered statistically significant.

## 3. Results

### 3.1. Influence of MXF on Plasma Biomarkers of Nephrotoxicity in Rat

Plasma levels of urea and creatinine have been considered suitable biomarkers of renal function.  Table 1 represents the plasma levels of urea and creatinine in rats following administration of MXF. Plasma urea level increased significantly (*P* < 0.05) by 25%, 35%, and 39% in the half-therapeutic, therapeutic, and double therapeutic dose groups. Plasma creatinine level also increased significantly by 46%, 78%, and 137%, respectively, in the MXF-treated animals.

### 3.2. Influence of MXF on Plasma Biomarkers of Hepatotoxicity in Rat

The effect of MXF on biomarkers of hepatotoxicity in rat is presented in Table 2. The plasma level of total bilirubin was significantly increased in the MXF-treated animals by 50%, 108%, and 133% compared to control. In a similar manner, activities of alkaline phosphatase (ALP), aspartate aminotransferase (AST), and alanine aminotransferase (ALT) were significantly increased (*P* < 0.05) in the plasma of MXF-treated animals by 9%, 15%, and 30%; 19%, 30%, and 40%; 82%, 129%, and 144%, respectively, when compared to control.

### 3.3. Influence of MXF on Plasma Lipid Profile of Rat

**Figure 2:**
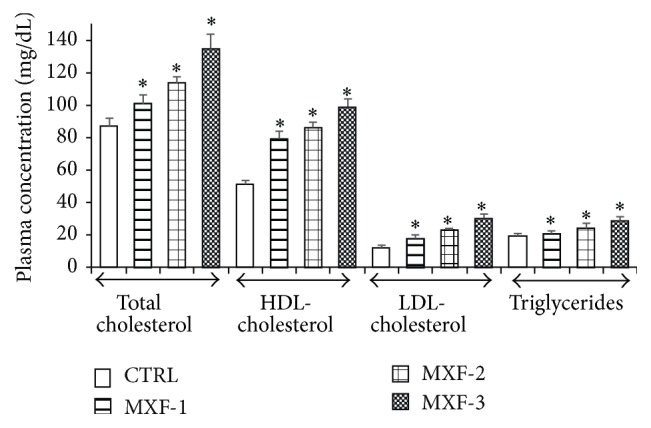
Influence of MXF on plasma lipid profile of rat. Values represent the mean ± SD of six replicates. ^*∗*^Significantly different from control (*P* < 0.05).

Figure 2 presents the effect of MXF on plasma lipid profiles of rats following treatment with MXF. Plasma levels of total cholesterol, HDL-cholesterol, LDL-cholesterol, and triglycerides increased significantly (*P* < 0.05) by 16%, 31%, and 55%; 54%, 68%, and 92%; 8%, 27%, and 50%; and 9%, 27%, and 54%, respectively, compared to control group.

### 3.4. Effect of MXF on the Hepatic Antioxidant Enzymes in Rat

Table 3 shows the effect of MXF for seven days on the activities of hepatic antioxidant enzymes of rat. There was a significant reduction in the activities of catalase, superoxide dismutase, and glutathione-S-transferase in the subtherapeutic, therapeutic, and double therapeutic groups by 18%, 32%, and 43%; 26%, 41%, and 60%; and 10%, 23%, and 38%, respectively, relative to control (*P* < 0.05).

### 3.5. Effect of MXF on the Hepatic Nonenzymatic Antioxidants in Rat

**Figure 3:**
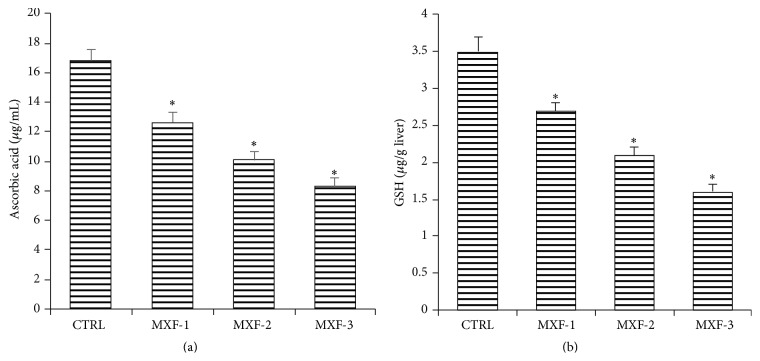
Influence of MXF on hepatic level of nonenzymatic antioxidants (a) ascorbic acid and (b) reduced glutathione levels in rat. Values represent the mean ± SD of six replicates. ^*∗*^Significantly different from control (*P* < 0.05).

The effects of seven-day MXF treatment of rats on hepatic nonenzymatic antioxidants, ascorbic acid, and reduced glutathione are shown in Figures 3(a) and 3(b), respectively. The levels of the ascorbic acid and GSH were significantly reduced (*P* < 0.05) by 25%, 40%, and 51% and 23%, 40%, and 51%, respectively, in the subtherapeutic, therapeutic, and double therapeutic MXF dose groups.

### 3.6. Effect of MXF on Hepatic Level of Lipid Peroxidation in Rat

**Figure 4:**
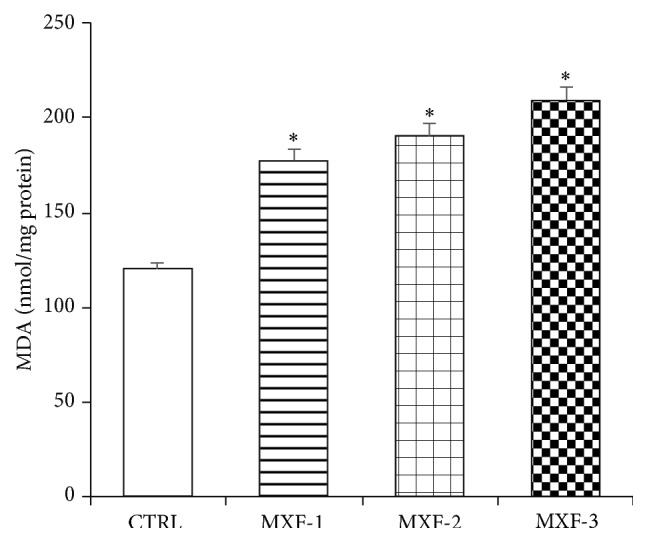
Influence of MXF on the hepatic malondialdehyde (MDA) level. Values represent the mean ± SD of six replicates. ^*∗*^Significantly different from control (*P* < 0.05).

The hepatic level of lipid peroxidation in rats following treatment with MXF is shown in Figure 4. Lipid peroxidation (MDA) level was significantly increased (*P* < 0.05) by 48%, 58%, and 74% in the liver of rats in the subtherapeutic, therapeutic, and double therapeutic MXF dose groups when compared to the control.

## 4. Discussion

In this study, we investigated the potential effect of three doses of moxifloxacin (MXF) on biomarkers of hepatotoxicity, nephrotoxicity, and oxidative stress in rat models. MXF is a fluoroquinolone antimicrobial agent with* in vitro *and* in vivo *activities against wide spectrum of Gram-negative and Gram-positive organisms [35]. Fluoroquinolones are the most widely used antibacterial agents today, thus prompting extensive studies of their potential adverse reactions in tissues* in vivo*. The plasma creatinine and urea levels have been widely used as suitable indicators of renal function in human and animal models [36, 37]. Creatinine is a breakdown product of creatine phosphate in muscle, while urea is major nitrogenous end product of protein and amino acid catabolism, produced by liver [38]. Data from this study suggest that MXF induced marked increase in plasma urea and creatinine levels. Creatinine and urea elevation of the plasma is an indication of abnormal renal function [39]. Plasma urea has been reported to increase in acute and chronic renal dysfunction and creatinine has been implicated in diseases such as acute kidney injury [39]. The observed increase in plasma urea and creatinine corroborates previous findings following administration of fluoroquinolone antibiotics [12, 40].

Our results indicate that administration of MXF for seven days resulted in elevated plasma total bilirubin (TBILI) and activities of ALP, ALT, and AST in experimental animals. Increase in plasma TBILI and ALP activity is known to be associated with hepatobiliary dysfunction which may have resulted from hepatobiliary injury and cholestasis [41]. Data from this study suggests that the dose dependent increase in plasma TBILI and ALP activity caused by the doses of MXF is an indication of hepatobiliary damage. Similar observation was reported by Fatai et al. [42] from studies on a fluoroquinolone antibiotic. Activities of ALT and AST are accepted marker of hepatocellular injury in human and animal models [43]. Elevated plasma ALT and AST may be linked with membrane leakage of the hepatocyte cytosolic contents which is reflected in significant elevation of the plasma of rats treated with different doses of MXF. This observation is in consonance with previous findings on other fluoroquinolones [44, 45].

As a physiological mechanism to prevent injury from reactive oxygen species (ROS), cells have developed strong antioxidant defence systems. Besides scavenger molecules such as glutathione (GSH), ascorbic acid (AA), or *α*-tocopherol, specific antioxidants enzymes such as catalase (CAT), superoxide dismutase (SOD), glutathione-S-transferase (GST), and glutathione peroxidase (GPx) also fulfil this task [46]. The activity or expression of these enzymes is known to be modulated by oxidative stress* in vivo *[47]. Data from this study indicate that the three doses of MXF administered altered both enzymatic and nonenzymatic antioxidant systems. The antioxidant enzymes CAT and SOD are part of the primary intracellular antioxidants defence mechanism against oxidative stress [47].

Superoxide radicals undergo dismutation by the action of SOD to hydrogen peroxide, while hydrogen peroxide formed is decomposed to water and molecular oxygen by CAT to prevent accumulation in the cell [48]. The decreased activities of SOD and CAT in the liver of the animals treated with the different doses of MXF could be due to the organ's response to an increased production of reactive oxygen species, as a result of exposure to the drugs and their metabolites. GST is a multifunctional enzyme and one of the key enzymes in drug metabolism, which is also known to play a vital role in redox balance in the cell [49]. It is involved in the biotransformation of xenobiotics, including drug detoxification leading to the elimination of toxic compounds [49]. The bactericidal action of quinolones is known to promote the generation of radicals most notably hydroxyl radicals in both Gram-negative and Gram-positive organisms as end product of drug metabolism [50, 51]. Furthermore, studies have demonstrated the capacity of quinolone drugs to generate free radicals and alter the activities of antioxidant enzymes* in vivo* [8, 52].

The hepatic level of GSH and AA is a measure of nonenzymatic antioxidant and cellular redox status of cells [48]. Studies have shown that the redox state of intracellular AA is closely influenced by the intracellular level of GSH [53]. Our studies showed that administration of MXF causes a depression of the overall redox status in the liver as indicated by data for AA and GSH. The observed reduction in hepatic GSH and AA content is in agreement with previous findings on fluoroquinolones [8, 42, 52].

Furthermore, data from this study indicated an alteration in levels of plasma total cholesterol, HDL-cholesterol, LDL-cholesterol, and triglycerides, with a concomitant increase in the hepatic malondialdehyde (MDA) level in the MXF-treated rats. The lipoproteins are the major transporters of both cholesterol and fatty acids* in vivo*. Low-density lipoprotein (LDL) is a vehicle to supply cholesterol all over the body in order to maintain cell viability and to provide cholesterol for various biosynthetic processes requiring cholesterol [54] while high density lipoprotein (HDL) plays a part in reverse cholesterol transport and also protects LDL from oxidation [55]. Lipoproteins and specifically the low-density lipoprotein (LDL) particles are susceptible to oxidation and peroxidation by prooxidants. Cholesterol and polyunsaturated fatty acids (PUFA) are the main components of LDL. Oxidation of lipoproteins is a lipid peroxidation process in which the unsaturated fatty acids (PUFAS) contents are transformed into lipid hydroperoxides, MDA, and other lipid peroxidation products [56]. Therefore, the more the amount of unsaturated lipids, the greater the level of lipid peroxidation and lipid peroxidation products formed. Increase in the level of MDA is a well-established biomarker of tissue damage [57]. The high level of lipid peroxidation following the administration of doses of MXF in the liver is a characteristic feature of certain fluoroquinolones [40, 44]. Depletion of the cell membrane antioxidant system is known to predispose membrane lipids to oxidation leading to accumulation of LPO products [58] including MDA as observed in this study. Previous studies have reported fluoroquinolone-induced increase in tissue MDA level [45, 59–61] and lipid peroxides [62]* in vivo*.

## 5. Conclusion

In conclusion, our results suggest that moxifloxacin altered renal and hepatic function as well as hepatic antioxidant defense systems of rat.

## Figures and Tables

**Table 1:** Influence of MXF on plasma biomarkers of renal toxicity in rat.

Treatment groups	Urea (mg/dL)	Creatinine (mg/dL)
Control	41.2 ± 3.8	0.37 ± 0.06
MXF-1	51.3 ± 2.1 (25%)^*∗*^	0.54 ± 0.03 (46%)^*∗*^
MXF-2	55.5 ± 3.5 (35%)^*∗*^	0.66 ± 0.05 (78%)^*∗*^
MXF-3	57.2 ± 2.6 (39%)^*∗*^	0.88 ± 0.07 (137%)^*∗*^

Values represent the mean ± SD of six replicates.  ^*∗*^Significantly different from control (*P* < 0.05); values in parenthesis represent % of increase.

**Table 2:** Influence of MXF on plasma biomarkers of hepatotoxicity in rat.

Treatment groups	TBILI (mg/dL)	ALP (U/L)	AST (U/L)	ALT (U/L)
CTRL	0.12 ± 0.02	239.8 ± 5.1	62.4 ± 2.1	21.6 ± 2.4
MXF-1	0.18 ± 0.03 (50%)^*∗*^	262.0 ± 4.2 (9%)^*∗*^	74.2 ± 2.8 (19%)^*∗*^	39.4 ± 2.7 (82%)^*∗*^
MXF-2	0.25 ± 0.02 (108%)^*∗*^	276.0 ± 6.5 (15%)^*∗*^	81.0 ± 3.7 (30%)^*∗*^	49.4 ± 3.1 (129%)^*∗*^
MXF-3	0.28 ± 0.04 (133%)^*∗*^	310.6 ± 8.3 (30%)^*∗*^	87.2 ± 4.6 (40%)^*∗*^	52.8 ± 3.3 (144%)^*∗*^

TBILI: total bilirubin; ALP: alkaline phosphatase; AST: aspartate aminotransferase; ALT: alanine aminotransferase.

Values represent the mean ± SD of six replicates. ^*∗*^Significantly different from control (*P* < 0.05); values in parenthesis represent % of increase.

**Table 3:** Influence of MXF on the activities of hepatic antioxidant enzymes in rat.

Treatment groups	CAT (*μ*mol H_2_O_2_ consumed/min/mg protein)	SOD (units/mg protein)	GST (nmol/min/mg protein)
CTRL	0.78 ± 0.05	13.8 ± 1.3	16.3 ± 1.7
MXF-1	0.64 ± 0.07 (18%)	10.2 ± 0.8 (26%)^*∗*^	14.6 ± 1.1 (10%)
MXF-2	0.53 ± 0.03 (32%)	8.1 ± 1.1 (41%)^*∗*^	12.5 ± 0.8 (23%)
MXF-3	0.44 ± 0.02 (43%)	5.5 ± 0.7 (60%)^*∗*^	10.1 ± 0.7 (38%)

Values represent the mean ± SD of six replicates. ^*∗*^Significantly different from control (*P* < 0.05); values in parenthesis represent % of decrease.
